# S7. NEOSENSITIZATION TO MULTIPLE DRUGS FOLLOWING DRUG REACTION WITH EOSINOPHILIA AND SYSTEMIC SYMPTOMS SYNDROME (DRESS)

**DOI:** 10.1093/schbul/sby018.794

**Published:** 2018-04-01

**Authors:** Moon Doo Kim, Won-Myong Bahk, Young Joon Kwon, Bo-Hyun Yoon, Kwanghun Lee, Beomwoo Nam, Eunsung Lim, Min-Kyu Song

**Affiliations:** 1 School of Medicine, Jeju National University; 2 Yeouido St. Mary’s Hospital, The Catholic University of Korea; 3 College of Medicine, Soonchunhyang University; 4 Naju National Hospital; 5 College of Medicine, Dongguk University; 6 School of Medicine, Konkuk University; 7 Shinsegae Hospital; 8 Yesan Mental Health Clinic

## Abstract

**Background:**

Drug Reaction with Eosinophilia and Systemic Symptoms (DRESS) syndrome is associated with severe skin eruptions, fever, hematological abnormalities, and multi-organ involvement. Although aromatic anticonvulsant drugs have been frequently associated with the manifestation of DRESS syndrome, its induction following treatment with nonaromatic anticonvulsants, such as valproate, has rarely been reported. Moreover, there are limited data regarding the development of neosensitization related to chemically unrelated drugs following an episode of DRESS syndrome.

**Methods:**

Here, a case of neosensitization to multiple drugs is described. The present case report describes a female patient who experienced neosensitization to amoxicillin, olanzapine, and quetiapine following the manifestation of DRESS syndrome induced by valproate.

**Results:**

A 50-year-old woman with a 15-year history of schizophrenia was being treated with lithium (1200 mg) and quetiapine (600 mg) about 1 month, but due to high lithium serum concentrations, the lithium was changed to valproate (600 mg). Seven days later, the patient developed a whole-body skin rash, facial edema, and hyperthermia. Laboratory tests revealed an abnormal white cell count (25.2 × 103 /μL with 6% eosinophils) and aspartate transaminase (AST) and alanine transaminase (ALT) concentrations of 2729 IU/L and 2749IU/L, respectively. At that time, the patient had no any other medical history including drug allergy. A diagnosis of DRESS syndrome due to valproate treatment was established by a consulting dermatologist. As a result, all medicines were discontinued because of severe hepatitis, and intravenous methylprednisolone (60 mg per day) was administered for 1 week. The skin rash, fever, and liver dysfunction progressively disappeared. After discharge, the patient was treated with quetiapine (200 mg). However, she became lost to follow up after 6 months. Approximately 3 years later, the patient was admitted to a local hospital for psychotic symptoms aggravation because she was not taken antipsychotics for 3 years. She treated with lithium (900 mg), sulpiride (600 mg), risperidone (2 mg), and quetiapine (100 mg) for 2 weeks. Additionally, the patient initiated treatment with amoxicillin for acute tonsillitis. On the first day of amoxicillin intake, she developed fever, diffuse erythematous macules on her trunk, and facial edema, and she was transferred to a general hospital via the emergency department. To control her psychotic symptoms she is prescribed olanzapine, haloperidol and quetiapine step by step but all these medications develop fever, skin rash and abnormal AST/ALT. Finally she was given amisulpiride which had not been previously prescribed. Within 2 months, the patient’s psychotic symptoms had gradually decreased and ultimately remitted.

**Discussion:**

To our knowledge, this is the first case report of neosensitization to multiple drugs after valproate-induced DRESS syndrome. A thorough search of Pubmed was performed to identify similar cases, which confirmed that no cases of hypersensitivity to amoxicillin or neosensitization to multiple drugs after a valproate-related DRESS episode have been reported. Furthermore, only two studies have reported possible neosensitization to amoxicillin following DRESS episodes induced by carbamazepine, and only one case reported neosensitization to amoxicillin following a DRESS episode induced by allopurinol.

